# Complete Genome Sequence of *Pantoea* sp. Strain CCBC3-3-1, an Antagonistic Endophytic Bacterium Isolated from a *Cotinus coggygria* Branch

**DOI:** 10.1128/MRA.01004-19

**Published:** 2019-09-19

**Authors:** Jianghong Zhou, Fei Xia, Shaochen Che, Li Zhong, Guofeng Zhang

**Affiliations:** a Beijing Key Laboratory of Ecological Function Assessment and Regulation Technology of Green Space, Beijing Institute of Landscape Architecture, Beijing, China; Broad Institute

## Abstract

Pantoea sp. strain CCBC3-3-1, having antagonistic activity against Verticillium dahlia, was isolated from Cotinus coggygria. We report the complete genome sequence of this strain determined by PacBio single-molecule real-time (SMRT) technology. The total genome size of CCBC3-3-1 is 5,159,767 bp, with a G+C content of 48.08%.

## ANNOUNCEMENT

The species in the genus of Pantoea are widely distributed in different ecological niches, and they are mainly associated with plants, either as pathogens or as epiphytes (1). Apart from that, some *Pantoea* species with biocontrol ability against plant pathogens have also been reported in recent years (2–4). In 2017, *Pantoea* sp. strain CCBC3-3-1 was isolated from a branch of *Cotinus coggygria* after surface sterilization with 75% (vol/vol) alcohol and 5% (wt/vol) sodium hypochlorite in Fragrant Hills Park in Beijing, China. Further analysis revealed that CCBC3-3-1 has strong growth inhibition activity on *Verticillium dahlia*, which is the causal agent of vascular wilt of *C. coggygria* (5). At the same time, the results of plant inoculation by stem injection (6) showed that this strain is nonpathogenic to *C. coggygria*, and it has high application potential for biocontrol of V. dahlia. To gain further understanding about this strain, complete genome sequencing was carried out in this study.

The stock bacterium strain CCBC3-3-1 was recovered on Luria broth (LB) solid medium by streaking (7), and single colonies were cultured in LB liquid medium in 2-ml microtubes and incubated at 30°C for 24 h with shaking. Genomic DNA was isolated using a Mag-MK bacterial genomic DNA extraction kit (Sangon Biotech, Shanghai, China), following the instructions of the manufacturer. The genome of CCBC3-3-1 was sequenced by Allwegene Technology Co., Ltd. (Beijing, China) using the PacBio RS platform with SMRTbell template prep kit 1.0 for library preparation. Around 3.3 Gb of data were obtained, with 600× average coverage. In total, 431,656 reads were generated, and the *N*_50_ read length is 8,707 bp. After filtered reads were assembled using SMRT Portal (8), genome assembly was done using Hierarchical Genome Assembly Process (HGAP) in SMRT Link v5.1.0 (9), and the coding sequences and tRNA and rRNA annotation were done by GeneMarkS v4.17 (10), tRNAscan-SE v1.3.1 (11), and rRNAmmer v1.2 (12), respectively. All software programs were used with default parameters.

The total size of the genome is 5,159,767 bp, with a G+C content of 48.08%, and it contains a 5,008,525-bp circular chromosome and three circular plasmids (plas1 [25,471 bp], plas2 [29,467 bp] and plas3 [96,304 bp]). A total of 5,013 coding DNA sequences (CDSs) were predicted. Of these, 4,240 CDSs could be assigned to a Clusters of Orthologous Groups (COG) number, and 326 CDSs were predicted secreted proteins. The most abundant COG category was amino acid transport and metabolism (432 proteins), followed by carbohydrate transport and metabolism (371 proteins), general function prediction only (367 proteins), transcription (313 proteins), and inorganic ion transport and metabolism (272 proteins). In addition, 145 noncoding RNAs, including 22 rRNAs, 85 tRNAs, and 38 small RNAs (sRNAs), were identified. The genomic information is shown in Table 1.

**TABLE 1:** Genome features of CCBC3-3-1

Feature	Value
Genome size (bp)	5,159,767
Chromosome size (bp)	5,008,525
plas1 size (bp)	25,471
plas2 size (bp)	29,467
plas3 size (bp)	96,304
No. of coding DNA sequences	5,013
No. of rRNAs (5S, 16S, 23S)	22
No. of tRNAs	85
No. of sRNAs	38
G+C content (%)	48.08
No. of GIs^*a*^	16
No. of genes assigned to COGs	4,240
No. of secreted proteins	326

a Gls, genomic islands.

### Data availability.

The complete chromosome sequence and three plasmid sequences have been deposited in GenBank under accession numbers CP034363 (chromosome), CP034364 (plas1), CP034365 (plas2), and CP034366 (plas3). The NCBI BioProject and BioSample accession numbers for this project are PRJNA506501 and SAMN10461917, respectively. The raw reads of sequenced genomic DNA were deposited in the SRA under accession number SRR9998537.
